# Cold‐Adapted Lipid A from *Polaribacter* sp. SM1127: A Study of Structural Heterogeneity and Immunostimulatory Properties

**DOI:** 10.1002/cbic.202500100

**Published:** 2025-04-21

**Authors:** Roberta Cirella, Emanuela Andretta, Luca De Simone Carone, Francesca Olmeo, Mei‐Ling Sun, Yu‐Zhong Zhang, Marcello Mercogliano, Antonio Molinaro, Alba Silipo, Flaviana Di Lorenzo

**Affiliations:** ^1^ Department of Chemical Sciences University of Naples Federico II Via Cinthia 4 80126 Naples Italy; ^2^ MOE Key Laboratory of Evolution and Marine Biodiversity Frontiers Science Center for Deep Ocean Multispheres and Earth System & College of Marine Life Sciences Ocean University of China 5 Yushan Rd, Shinan District Shandong Qingdao 266005 China; ^3^ Joint Research Center for Marine Microbial Science and Technology Shandong University Jinan, Licheng District, Jinan, Shanda S Rd, 27 Shandong Qingdao 250100 China; ^4^ State Key Laboratory of Microbial Technology Marine Biotechnology Research Center Shandong University Jinan, Licheng District, Jinan, Shanda S Rd, 27 Shandong Qingdao 250100 China; ^5^ CEINGE Istituto di Biotecnologie avanzate Via Gaetano Salvatore 486 80131 Naples Italy; ^6^ Department of Chemistry School of Science Osaka University 1‐1 Osaka University Machikaneyama Toyonaka Osaka 560‐0043 Japan

**Keywords:** innate immunities, lipid A, lipopolysaccharides, *Polaribacter*, structures to functions

## Abstract

*Polaribacter* sp. SM1127, a cold‐adapted marine Gram‐negative bacterium isolated from *Laminaria* in Arctic waters, plays a crucial role in nutrient cycling and biopolymer degradation in cold environments. Additionally, its exopolysaccharide (EPS) exhibits promising biotechnological potential, including antioxidant and wound‐healing properties. This study focuses on the isolation and characterization of lipid A, the glycolipid component of *Polaribacter* sp. SM1127 lipopolysaccharide (LPS), by bypassing full LPS extraction and working directly with the ethanol precipitation product containing both EPS and bacterial cells. Mass spectrometry analysis reveals significant structural heterogeneity in the lipid A, with variations in fatty acid chain length, branching, saturation, and hydroxylation. These features likely enable the bacterium to fine‐tune its response to fluctuating temperatures or other cold‐related environmental stresses, contributing to resilience in the Arctic Ocean ecosystem. Furthermore, immunological assays demonstrate that both LPS and EPS produced by *Polaribacter* sp. SM1127 induce weak Toll‐like receptor 4 activation and, in general, poorly stimulate the nuclear factor kappa‐light‐chain‐enhancer of activated B cells pathway, compared to *Escherichia coli* LPS. These findings suggest their potential as immunomodulatory agents, like vaccine adjuvants.

## Graphical abstract

This analysis reveals structural heterogeneity in the lipopolysaccharide lipid A from the cold‐adapted Gram‐negative bacterium *Polaribacter* sp. SM1127, with variations in fatty acid chain number, length, branching, saturation, and hydroxylation degree, likely aiding bacterial adaptation to cold environmental stressors. Lipopolysaccharide is shown to be a weak activator of Toll‐like receptor 4‐mediated pathway, highlighting its potential as an immunomodulatory molecule.

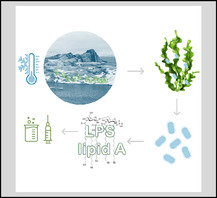

## 1 Introduction

The genus *Polaribacter* belongs to the family *Flavobacteriaceae* and comprises Gram‐negative marine bacteria displaying high versatility in cold and nutrient‐limited environments.^[^ ^1^ ^]^ Members of this genus are particularly abundant in polar and subpolar ecosystems, where they play key roles as degraders of biopolymers, such as proteins and polysaccharides.^[^ ^1^ ^]^ One such strain, *Polaribacter* sp. SM1127, was isolated from *Laminaria*, an important brown macroalgal species in Arctic coastal ecosystems. Indeed, *Polaribacter* sp. SM1127 represents a fascinating example of a marine bacterium able to interact with macroalgae despite the harsh conditions of the Arctic Ocean. It possesses enzymes capable of breaking down complex algal polysaccharides, such as laminarin and fucoidan, which are abundant in brown algae.^[^ ^2^ ^]^ These abilities not only enable the bacterium to flourish on the surface of *Laminaria* but also contribute to the biogeochemical cycling of carbon and other key nutrients in Arctic marine environments, thereby supporting the ecological dynamics of marine microbiomes.^[^ ^2^ ^]^ Recently, this *Polaribacter* strain has attracted particular interest for the biotechnological and pharmaceutical potential of its exopolysaccharide (EPS), which showed good antioxidant properties, excellent moisture‐retention ability, and the capability of promoting skin wound healing and preventing frostbite injury.^[^ ^3^, ^4^ ^]^

As a cold‐adapted bacterium, *Polaribacter* sp. SM1127 must exhibit a range of molecular adaptations, including modifications to its membrane composition, which enable it to manage osmotic and oxidative stress as well as the extreme temperature fluctuations. In this frame, the composition and structure of bacterial membranes are crucial for maintaining fluidity and functionality under reduced temperatures thus guaranteeing their survival in environments where other bacteria might fail. The lipopolysaccharide (LPS), as the main constituent of the outer membrane of Gram‐negative bacteria, is one of the principal molecules that undergo structural modifications that enhance membrane stability and protect bacteria from cold‐induced damage.^[^ ^5^ ^]^ LPS consists of a hydrophobic lipid A anchor, a core oligosaccharide, and a variable polysaccharide region termed O‐antigen. When the O‐antigen is absent, the terminology employed is rough‐type LPS or R‐LPS. The structural diversity of LPSs across different bacterial species enables adaptive strategies for surviving in a wide range of environments, including extreme conditions.^[^ ^6^ ^]^ In addition, modifications to the carbohydrate and lipid components of LPS, including the removal or addition of specific sugar residues, changes in the acylation of lipid A, or the introduction of alternative substituents, can profoundly alter properties of LPS‐expressing membranes.^[^ ^7^ ^]^

On one hand, the ability to modify LPS structures and analyze the resulting changes provides valuable insight into how cold‐adapted bacteria manage to survive and thrive in extreme conditions. On the other hand, it is largely known that the LPS is able to activate an immune inflammatory response in mammals by interacting, among others, with the innate immunity receptorial complex made up of myeloid differentiation protein‐2 (MD‐2) and Toll‐like receptor 4 (TLR4).^[^ ^5^, ^8^, ^9^ ^]^ The immunological activity of LPS is strongly linked to its chemical structure, which, in the case of pathogen‐derived LPS, triggers the production of pro‐inflammatory cytokines and exhibits distinct “immunostimulatory” chemical characteristics. However, any structural modifications to the LPS can lead to changes in its inflammatory capacity, influencing both the quantity and the nature of cytokines released by immune cells.^[^ ^5^ ^]^ Contextually, we have previously reported about LPS from extremophilic bacteria able to only poorly activate the TLR4‐mediated immune response,^[^ ^10^, ^11^, ^12^, ^13^, ^14^, ^15^, ^16^ ^]^ thus paving the way to their possible use as immunomodulatory compounds in the perspective of drug synthesis and development.

As the main membrane component affected by environmental pressures and, at the same time, the portion of LPS recognized by TLR4/MD‐2, here we report an in‐depth analysis of the chemical structure of lipid A from *Polaribacter* sp. SM1127 LPS. To this aim, we have isolated and characterized the lipid A bypassing the extraction procedure of the full LPS and by working on the product of ethanol precipitation performed to obtain EPS, which contained both the EPS fraction and bacterial cells, as previously described.^[^ ^17^ ^]^ Through this investigation, we aim at enhancing our understanding of microbial adaptation to cold marine environments and provide further evidence of the significance of structure–function studies of glycans and glycoconjugates from extreme environments. These insights could help identify novel molecules for use in or as inspiration for the development of adjuvants or immunotherapeutic agents.

## 2 Results and Discussion

### 2.1 Isolation and Chemical Analyses of the LPS from *Polaribacter* sp. SM1127

**Figure 1:**
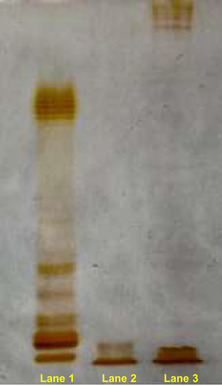
Sodium dodecyl sulphate–polyacrylamide gel electrophoresis (SDS–PAGE) after silver staining of *Polaribacter* sp. SM1127 R‐LPS obtained upon enzymatic digestion of UCF_pc_ (Lane 2). Crude EPS (EPS‐EtOH) was also run in lane 3 and clearly showed the presence of both EPS (upper band) and R‐LPS (lower band). S‐LPS from *E. coli* O111:B4 (Lane 1) was used as a benchmark.

To isolate and characterize the lipid A from *Polaribacter* sp. SM1127 LPS, two approaches were employed (Figure S1, Supporting Information). First, an aliquot of ethanol precipitation product (EPS‐EtOH) containing both EPS and bacterial cell remnants underwent mild acid hydrolysis with acetate buffer, followed by centrifugation to isolate the lipid A in the water‐insoluble fraction (LipA_abh_). In the second approach, EPS‐EtOH was ultracentrifuged to separate bacterial cells (which formed the precipitate) from the EPS (found in the supernatant). Both fractions were then lyophilized and enzymatically digested. The precipitate from ultracentrifugation (UCF_pc_) was analyzed by sodium dodecyl sulphate–polyacrylamide gel electrophoresis (SDS–PAGE) with silver nitrate staining, which confirmed the presence of LPS in this fraction and indicated its “rough” nature, as evidenced by a gel band typical of low‐molecular‐mass R‐LPS, lacking the O‐antigen (**Figure** **1**). Following the removal of phospholipids using sequential chloroform–methanol and chloroform–methanol–water washes, UCF_pc_ underwent a small‐scale extraction procedure to isolate the lipid A fraction (LipA_sse_). Both LipA_sse_ and LipA_abh_ were then subjected to compositional analysis to determine their fatty acid and monosaccharide content. In particular, aliquots of LipA_sse_ and LipA_abh_ underwent methanolysis, followed by acetylation to analyze the fatty acids as methyl esters and to identify the sugar components of the lipid A backbone. The analysis revealed an extremely heterogeneous fatty acid composition, which was qualitatively similar for both LipA_sse_ and LipA_abh_ (**Table** **1**), and as expected showed the occurrence of the acetylated methyl glycoside derivative of 2‐amino‐2‐deoxy‐D‐glucose (D‐glucosamine). This, along with the fatty acid analysis, provided crucial information for the elucidation of the lipid A structure, which was achieved by matrix‐assisted laser desorption/ionization ‐ time of flight (MALDI‐TOF) mass spectrometry (MS) and MS/MS analysis of both LipA_sse_ and LipA_abh_.

**Table 1:** Fatty acid content of LipA_sse_ and LipA_abh_ isolated from *Polaribacter* sp. SM1127.

Fatty acid componenta)
*i*13:0
13:0
*i*14:0
14:0
14:0(3‐OH)
*i*15:1
*i*15:0
15:0 (*a*15:0)
15:1
15:0
*i*15:0(3‐OH)
*a*15:0(3‐OH)
15:0(3‐OH)
16:1
16:0
*i*16:0(3‐OH)
16:0(3‐OH)
*a*17:1
17:1
17:0
*a*17:0(3‐OH)

a) Both lipid A fractions showed the same fatty acid composition. All 3‐hydroxy fatty acids displayed an (*R*) configuration. The position of the double bond or the stereochemistry of the unsaturated acyl chains remain to be identified. The stereochemistry of anteiso‐branched acyl chains is tentatively given as (*S*) being predominant in bacteria; however, it remains to be confirmed. “*i*” stands for iso and “*a*” stands for anteiso.

### 2.2 MALDI‐TOF MS and MS/MS Analysis of the Lipid A Isolated from *Polaribacter* sp. SM1127

**Figure 2:**
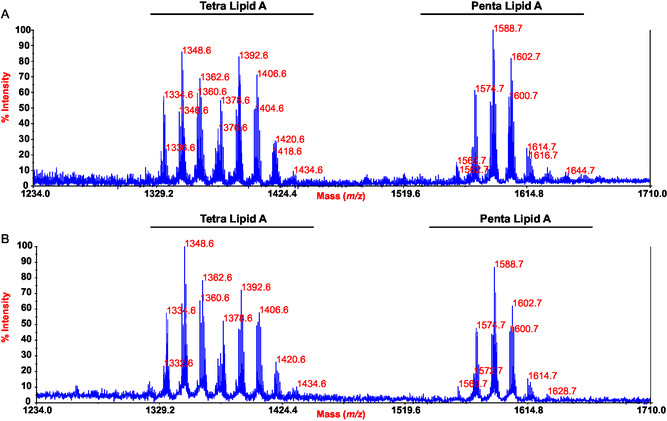
Reflectron MALDI‐TOF MS spectra, recorded in negative‐ion polarity, of A) LipA_sse_ and B) LipA_abh_. Lipid A species are labeled as Tetra LipA and Penta LipA on the basis of the degree of acylation.

Reflectron MALDI‐TOF mass spectra, acquired in negative ion mode, for both LipA_sse_ and LipA_abh_ are shown in **Figure** **2**. The spectra were nearly identical, confirming the effectiveness of the methods used to isolate lipid A directly from EPS‐EtOH. In both MS spectra, two major groups of peaks in ≈1:1 ratio were identified, corresponding to deprotonated [M—H]^−^ mono‐phosphorylated penta‐acylated (around *m/z* 1588) and tetra‐acylated lipid A species (around *m/z* 1348/1392). The high structural variability in *Polaribacter* sp. SM1127 lipid A was evident, as proven by the presence of peaks differing by 14 amu, indicative of variations in the length of the acyl chains. Additional complexity was observed due to the presence of unsaturated fatty acids, which caused peaks to differ by 2 amu. As an example, in the *m/z* range of 1560.7–1644.7 (Figure 2), the peak at *m/z* 1602.7 was attributed to a penta‐acylated lipid A structure consisting of the typical glucosamine disaccharide backbone with a single phosphate group and bearing three 15:0(3‐OH) [or *i*15:0(3‐OH) or *a*15:0(3‐OH)], >one 14:0 [or *i*14:0] and one 17:0 (**Table** **1** and **2**). For the sake of simplicity, hereafter, we will refer to the acyl chains without specifying their branching; however, they may occur in iso‐ or anteiso‐ forms, as detailed in the compositional analysis (Table 1). In the mass region *m/z* 1334.6–1434.6 (Figure 2), a heterogenous blend of tetra‐acylated lipid A species was identified differing for the acyl chains length and degree of hydroxylation and unsaturation. As example, the peak at *m/z* 1348.6 was matched with a mono‐phosphorylated lipid A species carrying one 15:0(3‐OH), one 14:0(3‐OH), one 17:0, and one 14:0, while the peak at 1392.6 carries one phosphate and three 15:0(3‐OH) and one 17:0 (Table 2).

**Table 2:** Main ion peaks detected in the MALDI‐TOF MS spectra reported in Figure 2, the predicted masses, and the proposed interpretation of the fatty acids and phosphate (P) on the lipid a diglucosamine backbone.

Predicted mass [Da]a)	Observed ion peaks [*m/z*]	Acyl substitution	Proposed composition
1615.18 (LA1)	1614.7 (LA1)	Penta‐acyl	HexN^2^, P, [16:0(3‐OH)]^2^ [15:0(3‐OH)] (16:1) (14:0)
1615.18 (LA2)	1614.7 (LA2)	Penta‐acyl	HexN^2^, P, [15:0(3‐OH)]^2^ [16:0(3‐OH)] (16:1) (15:0)
1615.18 (LA3 and LA4)	1614.7 (LA3 and LA4)	Penta‐acyl	HexN^2^, P, [15:0(3‐OH)]^2^ [16:0(3‐OH)] (17:1) (14:0)
1603.18	1602.7	Penta‐acyl	HexN^2^, P, [15:0(3‐OH)]^3^ (17:0) (14:0)
1589.18	1588.7	Penta‐acyl	HexN^2^, P, [15:0(3‐OH)]^2^ [14:0(3‐OH)] (17:0) (14:0)
1407.0 (LA1)	1406.6 (LA1)	Tetra‐acyl	HexN^2^, P, [16:0(3‐OH)]^2^ [17:0(3‐OH)] (14:0)
1407.0 (LA2)	1406.6 (LA2)	Tetra‐acyl	HexN^2^, P, [15:0(3‐OH)] [16:0(3‐OH)] [17:0(3‐OH)] (15:0)
1392.96	1392.6	Tetra‐acyl	HexN^2^, P, [15:0(3‐OH)]^3^ (17:0)
1362.97	1362.6	Tetra‐acyl	HexN^2^, P, [15:0(3‐OH)]^2^ (17:0) (14:0)
1348.96	1348.6	Tetra‐acyl	HexN^2^, P, [15:0(3‐OH)] [14:0(3‐OH)] (17:0) (14:0)

a) The observed masses are compared to the calculated monoisotopic mass (predicted mass, Da) of each ion based on the proposed lipid A chemical structures.

**Figure 3:**
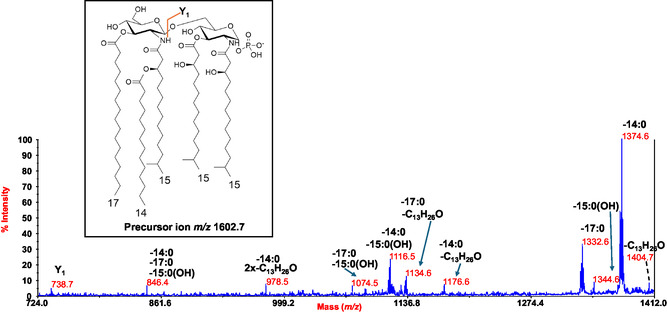
Negative‐ion MALDI MS/MS spectrum of precursor ion at *m/z* 1602.7, a representative ion peak of the cluster ascribed to mono‐phosphorylated penta‐acylated lipid A species detected for *Polaribacter* sp. SM1127 LipA_sse_. The major fragment assignments are displayed in the spectrum, accompanied by a proposed structural model illustrated in the inset. The nature of the branched acyl chains (iso) within the lipid A structure is included for descriptive purposes. Additionally, peaks resulting from the loss of C_13_H_26_O (198 mass units), caused by rearrangements in the N‐linked acyl chains with a free hydroxyl group, are also reported.

To locate the phosphate group and acyl chains on the disaccharide backbone, negative‐ion MALDI‐TOF MS/MS was employed on several peaks. The MS/MS spectrum of the precursor ion at *m/z* 1602.7 (**Figure** **3**) showed prominent peaks at *m/z* 1374.6 and 1332.6, generated by the loss of 14:0 and 17:0, respectively. A less intense peak corresponding to the loss of a 15:0(3‐OH) was also observed at *m/z* 1344.6. Additional peaks were detected at *m/z* 1074.5 and 1116.5, resulting from the sequential loss of a primary 15:0(3‐OH) and a 17:0(*m/z* 1074.5) or a 14:0(*m/z* 1116.5) unit. Since no ions attributable to the loss of a whole unit comprising 15:0(3‐OH) and either 14:0 or 17:0 were observed, it was hypothesized that these non‐hydroxylated fatty acids were not part of acyloxyacyl moieties. However, the merging of several minor peaks provided additional evidence that guided the positioning of all acyl chains. Specifically, peaks detected at *m/z* 1134.6 and *m/z* 1176.6 were matched with fragments devoid of 17:0 or 14:0, plus 198 amu, respectively. The loss of 198 amu was explained by a rearrangement that can only occur when the N‐linked acyl chains do not carry secondary acyl groups, i.e., they have a free 3‐OH group. Indeed, an enamine to imine tautomerization followed by six‐membered‐ring‐based rearrangement can produce the loss of a C_13_H_26_O neutral fragment (198 amu) from each primary amide‐linked 15:0(3‐OH) having a free 3‐OH group.^[^ ^18^ ^]^ Since these rearrangements were observed when also 17:0 or 14:0 were absent, one can speculate that both these fatty acids were linked to the two N‐linked 15:0(3‐OH). However, the observation of the peak at *m/z* 1404.7, generated by the sole loss of 198 amu, supported the hypothesis that only one of the two fatty acids (17:0 or 14:0) was attached to a primary amide‐bound 15:0(3‐OH). This was further demonstrated by the detection of the Y‐type ion^[^ ^19^ ^]^ at *m/z* 738.7, originating from the cleavage of the glycosidic bond between the two glucosamines. This was crucial for placing two 15:0(3‐OH) on the reducing glucosamine, along with the phosphate group, and simultaneously locating the 17:0, 14:0, and another 15:0(3‐OH) on the nonreducing glucosamine. Finally, the detection of the peak at *m/z* 978.5, corresponding to the loss of two C_13_H_26_O neutral fragments (198 + 198 amu) plus one 14:0, helped identify the 17:0 as a primary ester‐bound fatty acid on the nonreducing glucosamine, with the 14:0 identified as acyloxyacylamide moiety of the same glucosamine.

**Figure 4:**
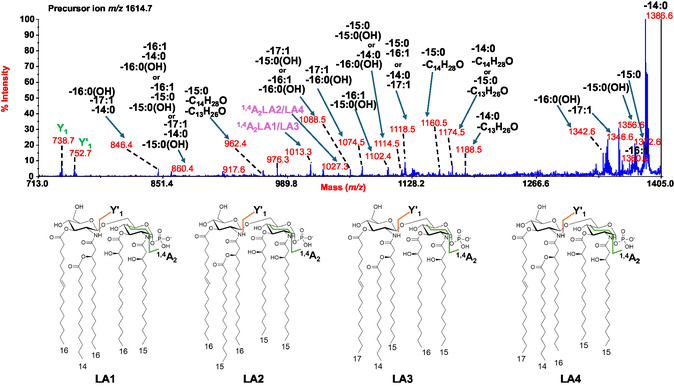
Negative‐ion MALDI MS/MS spectrum of precursor ion at *m/z* 1614.7, another representative peak of the cluster that matched with mono‐phosphorylated penta‐acylated lipid A species and found for *Polaribacter* sp. SM1127 LipA_sse_ and LipA_abh_. The key fragment assignments are provided. Below the spectrum, the structures of the four isobaric forms are shown, with fatty acids represented in their unbranched form for simplicity. Peaks originated from the loss of C_13_H_26_O (198 mass units) and/or C_14_H_28_O (212 mass units) have been also indicated.

To gain insights into lipid A species displaying unsaturated acyl chains, precursor ion at *m/z* 1614.6 was chosen as a representative and underwent MS/MS investigation (**Figure** **4**). This analysis was crucial in bringing to light the intrinsic additional heterogeneity of *Polaribacter* sp. SM1127 lipid A, which was justified by its fatty acid content (Table 1). Indeed, at least four isobaric lipid A species were assigned to ion at *m/z* 1614.7 (LA1–LA4, Figure 4) differing for the length of the acyl chains. Briefly, the spectrum clearly displayed main peaks at *m/z* 1386.6, *m/z* 1372.6, *m/z* 1360.6, *m/z* 1356.6, *m/z* 1346.6, and *m/z* 1342.6 attributed to ions derived by the loss of 14:0, 15:0, 16:1, 15:0(3‐OH), 17:1, and 16:0(3‐OH), respectively. Four main observations were key to define the structure of each lipid A species: 1) the examination of peaks matching with fragments originated by the loss of combinations of the aforementioned acyl chains; 2) the detection of ions generated by the loss of acyl chains plus a neutral fragment derived from enamine to imine tautomerization; 3) the assignment of Y‐type ions (*m/z* 738.7 and *m/z* 752.7) to counterprove the nature of the fatty acids on the reducing glucosamine unit and, specularly, of those on the nonreducing one; 4) the identification of ^1,4^A_2_ cross‐ring fragmentation^[^ ^19^ ^]^ derived ions at *m/z* 1013.3 and *m/z* 1027.3, which finally confirmed the structural assignments. Overall, the structures identified for precursor ion at *m/z* 1614.7, summarized in Figure 4, comprise primary ester‐bound 17:1 or 16:1 on the nonreducing glucosamine, primary ester‐bound 16:0(3‐OH) or 15:0(3‐OH) on the reducing unit, primary amide‐linked 16:0(3‐OH) and/or 15:0(3‐OH) on the nonreducing unit while always 15:0(3‐OH) was found on the reducing one, and 14:0 or 15:0 as secondary acyl substituents in an acylooxyacylamide moiety of the nonreducing residue.

**Figure 5:**
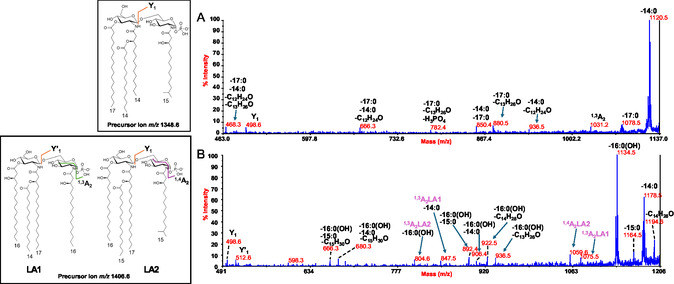
Negative‐ion MALDI MS/MS spectra of precursor ions at A) *m/z* 1348.6 and B) *m/z* 1406.6, chosen as representatives of the heterogeneous cluster ascribed to mono‐phosphorylated tetra‐acylated lipid A species. In the inset, it is reported the proposed structure, where the iso‐branching of the fatty acids is tentative. Peaks originated from the enamine to imine tautomerization and subsequent rearrangement have been also reported.

Likewise, the structure of tetra‐acylated lipid A species detected at *m/z* 1348.6 and *m/z* 1406.6 were also defined (**Figure** **5**). The analysis of the MS/MS spectrum of the latter precursor ion highlighted once again the existence of two isobaric species differing for the length of the secondary fatty acid decorating the nonreducing glucosamine (14:0 or 15:0) and of the primary N‐linked acyl chain on the reducing one [15:0(3‐OH) or 16:0(3‐OH)]. In conclusion, integrating data from chemical analyses and MS/MS‐based structural studies, we show that *Polaribacter* sp. SM1127 synthesizes a remarkably heterogeneous mixture of tetra‐ and penta‐acylated lipid A species. These species feature the typical glucosamine disaccharide backbone decorated by one phosphate on the reducing glucosamine unit and carrying acyl chains of various lengths and branching degree, also experiencing non‐hydroxylated and/or unsaturated acyl chains as primary ester‐bound moieties of the nonreducing glucosamine.

### 2.3 Immunostimulatory Properties of *Polaribacter* sp. SM1127 LPS and EPS

**Figure 6:**
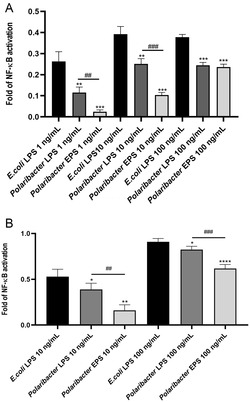
A) Stimulation of HEK‐Blue hTLR4 cells. SEAP levels (OD 620 nm) following stimulation with various concentrations (1–10–100 ng mL^−1^) of *E. coli* O111:B4 LPS (*E. coli* LPS), *Polaribacter* sp. SM1127 LPS (*Polaribacter* LPS), or EPS (*Polaribacter* EPS) were assessed by Quanti‐blue assay. B) Stimulation of PMA‐differentiated THP1‐Blue NF‐κB cells. SEAP levels (OD 620 nm) following stimulation with 10 or 100 ng mL^−1^ of *E. coli* LPS, *Polaribacter* LPS, or EPS were assessed by Quanti‐blue assay as well. Data are normalized over unstimulated cells and shown as mean ± standard deviation. Significant differences between *Polaribacter* LPS or EPS and *E. coli* LPS (*p‐value < 0.05, **p‐value < 0.01, ****p‐value < 0.0001 vs *E. coli* LPS) and between *Polaribacter* LPS and EPS (^##^ *p*‐value < 0.01, ^###^ *p*‐value < 0.001) were evaluated by unpaired *t*‐test.

The peculiar structural diversity of *Polaribacter* sp. SM1127 lipid A sparked our interest in investigating the immunostimulatory activity of its LPS. Activation of TLR4, the main receptor able to recognize lipid A, triggers the Nuclear Factor kappa‐light‐chain‐enhancer of activated B cells (NF‐kB) pathway which in turn is responsible for regulating the expression of various pro‐inflammatory genes and therefore it represents a key mediator of the inflammatory response.^[^ ^20^ ^]^ As such, we used human embryonic kidney‐blue (HEK‐Blue) tohoku hospital pediatrics‐1 (THP‐1) cells stably transfected with human TLR4 and MD‐2/CD14 featuring an NF‐κB/AP‐1‐inducible secreted embryonic alkaline phosphatase (SEAP) reporter gene to evaluate the TLR4‐dependent NF‐κB response upon stimulation with LPS. In these experiments, EPS, which underwent additional steps of purification to remove traces of bacterial cells, was also tested and its activity compared to that of the LPS. We stimulated HEK‐Blue hTLR4 cells with various concentrations (1–10–100 ng mL^−1^) of both *Polaribacter* sp. SM1127 LPS and EPS. HEK‐Blue hTLR4 cells stimulated with *Escherichia coli* LPS (1–10–100 ng mL^−1^) were used as a positive control, while unstimulated cells were used as a negative control. Both *Polaribacter* sp. SM1127 LPS and EPS treatment resulted in a significantly lower TLR4 activation than *E. coli* LPS at all tested concentrations (*Polaribacter* LPS vs *E. coli* LPS *p* < 0.01 at 1 and 10 ng mL^−1^, *p* < 0.001 at 100 ng mL^−1^; *Polaribacter* EPS vs *E. coli* LPS *p* < 0.001 at all concentrations, **Figure** **6**a). Interestingly, treatments with 1 and 10 ng mL^−1^ of EPS were associated with an even weaker activation of TLR4 compared to *Polaribacter* LPS used at the same concentrations (*Polaribacter* EPS vs *Polaribacter* LPS *p* < 0.01 at 1 and 10 ng mL^−1^, Figure 6a). No significant differences were detected between *Polaribacter* sp. SM1127 LPS and its EPS at 100 ng mL^−1^.

Although we cannot fully exclude the presence of trace amounts of bacterial debris in the EPS preparations, which may contribute to the observed TLR4‐mediated signaling at 100 ng mL^−1^, this finding is in line with other studies reporting that certain polysaccharides can activate TLR4 even at concentrations lower than those typically required for such molecules (i.e., 1–500 μg mL^−1^).^[^ ^21^, ^22^ ^]^ To further investigate the immunological properties of LPS and EPS in real immune cells, we evaluated their effect on Tohoku Hospital Pediatrics‐1 (THP1)‐Blue NF‐κB cells. Once differentiated into macrophages, Quanti blue assay was conducted upon stimulation of cells with LPS or EPS from *Polaribacter* at 10 and 100 ng mL^−1^, as well as with *E. coli* LPS used again as positive control. THP1 cells stimulated with *Polaribacter* LPS (10 and 100 ng mL^−1^) showed reduced NF‐kB activation compared to that observed for the same concentrations of *E. coli* LPS (*Polaribacter* LPS vs *E. coli* PS *p* < 0.05 at both concentrations, Figure 6b). Likewise, EPS behaved as an even weaker activator compared to *E. coli* LPS (*Polaribacter* LPS vs EPS *p* < 0.01 at 10 ng mL^−1^ and *p* < 0.0001 at 100 ng mL^−1^, Figure 6b). Finally, *Polaribacter* sp. SM1127 LPS and EPS exhibited different immunostimulatory capacity also in THP‐1 as NF‐kb activation was significantly lower in THP1 cells treated with EPS compared to those stimulated with LPS at both concentrations used (*Polaribacter* EPS vs *Polaribacter* LPS *p* < 0.01 at 10 ng mL^−1^, *p* < 0.001 at 100 ng mL^−1^, Figure 6b).

## 3 Conclusion

Recently studied for the potential biotechnological applications of its EPS in the food, cosmetic, pharmaceutical, and biomedical fields^[^ ^3^, ^4^ ^]^ *Polaribacter* sp. SM1127 was never analyzed for its LPS content. In this study, we aimed at isolating the lipid A fraction of the R‐LPS synthesized by *Polaribacter* sp. SM1127, using the cold ethanol precipitation product (EPS‐EtOH) that was previously obtained to isolate the EPS material. Our approach (outlined in Figure S1, Supporting Information), which we previously successfully adopted on another extremophilic bacterium,^[^ ^17^ ^]^ involved a mild acid hydrolysis of the EPS‐EtOH (LipA_abh_) and ultracentrifugation followed by lipid A small‐scale extraction from the resulting pellet (LipA_sse_). The comprehensive structural analysis of the lipid A fraction using MALDI‐TOF MS and MS/MS techniques has revealed significant heterogeneity in the lipid A species, highlighting their complex composition and variations. Both LipA_abh_ and LipA_sse_ exhibited nearly identical reflectron MALDI‐TOF mass spectra, validating the effectiveness of our isolation methods and confirming the direct extraction of lipid A from EPS‐EtOH.

The remarkable structural heterogeneity of *Polaribacter* sp. SM1127 lipid A has important implications for understanding its function in cold adaptation. The presence of both tetra‐ and penta‐acylated lipid A species and the variability in acyl chain length and hydroxylation points to a sophisticated mechanism of lipid A biosynthesis that likely plays a role in adapting the bacterial membrane to cold environments by enhancing membrane flexibility and fluidity to maintaining its proper function under low temperatures.^[^ ^23^, ^24^ ^]^ For instance, the introduction of unsaturated acyl chains into lipid A, observed in several cold‐adapted bacteria such as *Pseudoalteromonas tetraodonis*, *Psychromonas arctica* and *Psychromonas marina*, and *Psychrobacter cryohalolentis*, among many others,^[^ ^25^, ^26^ ^]^ is considered an adaptive strategy to ensure membrane fluidity at low temperatures, where more rigid, saturated lipids would otherwise impair membrane function.^[^ ^24^, ^25^, ^26^ ^]^ Interestingly, the presence of both saturated and unsaturated, non‐hydroxylated acyl chains as primary fatty acids found in some lipid A species, such as *Echinicola* species,^[^ ^12^ ^]^ is relatively rare and adds a distinctive aspect to the lipid A profile of *Polaribacter* sp. SM1127. As example, unsaturated fatty acids are more often found as secondary substituents, whereas the presence of these fatty acids as primary acyl chains of lipid A suggests a specialized adaptation to cold environments exploited by *Polaribacter* sp. SM1127. This incorporation might further enhance membrane flexibility and permeability, which could be particularly advantageous in extreme cold conditions where maintaining efficient nutrient uptake and ion transport is vital for survival. Likewise, another intriguing aspect of the lipid A heterogeneity observed in *Polaribacter* sp. SM1127 is the inclusion of branched acyl chains, which is commonly observed in marine bacteria experiencing highly mutable habitats, and is thought to contribute to membrane stability and fluidity by causing a greater disturbance of the packing order of the lipid chains within the membrane.^[^ ^27^, ^28^, ^29^, ^30^ ^]^ Overall, all these unique structural features in its lipid A enable *Polaribacter* sp. SM1127 to maintain a constantly dynamic membrane structure which is crucial for surviving and thriving in the fluctuating and extreme conditions of the Arctic Ocean.

From another perspective, due to the structure‐dependent capability of LPS of inducing a TLR4‐mediated immune response, such a significant structural diversity of *Polaribacter* sp. SM1127 lipid A could have functional implications for how the bacterium interacts with this receptor.^[^ ^5^, ^9^, ^31^, ^32^ ^]^ Indeed, our immunological assays demonstrated that *Polaribacter* sp. SM1127 LPS induces considerably reduced TLR4 activation compared to *E. coli* LPS, a potent TLR4 agonist and pro‐inflammatory molecule. This suggests that the unique structural features of its lipid A, particularly variations in acyl chain number and length, play a key role in modulating the bacterium ability to trigger immune responses. Typically, LPSs with hypo‐acylated lipid A species (e.g., penta‐ and tetra‐acylated forms) induce a milder TLR4 activation compared to hexa‐acylated lipid A species, such as those found in *E. coli*.^[^ ^5^ ^]^ This diminished activity is further attributed to the presence of only one phosphate group on the lipid A, which is commonly associated with a reduced immunostimulatory capacity compared to lipid A forms where two or more phosphate groups are found.^[^ ^33^ ^]^ In line with these findings, THP‐1 cells treated with *Polaribacter* sp. SM1127 LPS exhibited attenuated NF‐kB pathway activation, reinforcing the notion that its lipid A has a lower immunostimulatory potential compared to *E. coli* LPS. This weaker response aligns with previous research indicating that extremophilic bacterial LPS often exhibit reduced immunogenicity compared to more “conventional” LPS sources.^[^ ^6^, ^10^, ^11^, ^12^, ^13^, ^14^, ^15^, ^34^ ^]^ This further strengthens the importance of studying LPS from bacteria experiencing extreme environments as this immunological characteristic is particularly advantageous in applications where the modulation of immune responses is sought, such as in the development of immunotherapeutics or vaccine adjuvants that require a controlled, balanced immune response, reducing the risk of overactive reactions that can lead to adverse effects.^[^ ^35^ ^]^ Overall, the unique structural features of *Polaribacter* sp. SM1127 lipid A, combined with its potential to modulate immune responses, underscore the ecological and biotechnological significance of this cold‐adapted bacterium. Future studies should aim to further elucidate the detailed mechanisms by which this LPS interacts with the host immune system thus potentially paving the way to new biomolecules with tailored immune‐modulatory functions to be used for future applications in biomedicine.

Finally, in this study, we also tested *Polaribacter* sp. SM1127 EPS, which was known for its wound healing and cryoprotectant properties, but whose immunostimulatory potential was never analyzed. Our results showed that the EPS was an even weaker activator of the NF‐kB pathway compared to *Polaribacter* sp. SM1127 LPS and, obviously, of *E. coli* LPS. This observation further supports the previously proposed good potential of this EPS in the pharmaceutical area. Indeed, the reduced inflammation and faster healing observed in previous experiments suggested that this compound may mitigate the damage caused by freezing temperatures and facilitate skin recovery.^[^ ^3^ ^]^ The fact that it appears to have only weak immunostimulatory effects, specifically with respect to the NF‐kB pathway, is an additional value of this EPS that could be an advantageous feature in pharmaceutical applications, especially when considering the balance between promoting tissue repair and avoiding excessive immune activation.

All in all, these findings emphasize the promise of *Polaribacter* sp. SM1127 EPS and lipid A in developing novel biomolecules for immune‐modulatory and tissue‐protective applications.

## 4 Experimental Section

4.1

4.1.1

##### Isolation and Purification of the R‐LPS and Lipid A Fraction from *Polaribacter* sp. SM1127

To streamline the procedure, R‐LPS/lipid A extraction was performed directly on the ethanol precipitation product (EPS‐EtOH), which contained residual cell debris, thus bypassing the need for full LPS extraction and reduce the minimum sample input needed, which is particularly advantageous when working with bacterial strains that yield low biomass, as in the case of extremophiles. Briefly, *Polaribacter* sp. SM1127 was grown in a basal marine medium at 15 °C and 200 rpm for 5 days to produce EPS. Then, an aliquot of the fermentation broth was mixed with two volumes of chilled absolute ethanol to precipitate the EPS, and then the mixture was lyophilized.^[^ ^3^, ^4^ ^]^ The resulting lyophilized precipitate was dissolved in deionized water (3%, w/v) and treated with 15 units mL^−1^ of compound protease (Gold Wheat, China) at 50 °C and 120 rpm for 5 h to remove proteins. After an additional precipitation step with cold absolute ethanol, the crude EPS (EPS‐EtOH), containing both EPS and residual bacterial cells, was resuspended in distilled water and lyophilized.^[^ ^3^, ^4^ ^]^ To separate cells from the EPS, 40 mg of the dried EPS‐EtOH was subjected to ultracentrifugation (208,000 × g, 4 °C for 16 h). The supernatant, mainly containing the EPS, and the pellet (UCF_prec_), containing the bacterial cells, were collected separately, lyophilized, and analyzed using SDS–PAGE after enzymatic digestion.^[^ ^36^ ^]^ In brief, 1 mg of each dried sample was diluted in 1 mL of a buffer solution (0.5 M Tris HCl [pH 6.8], glycerol [20% vol/vol], mercaptoethanol [4% vol/vol], SDS [4% wt/vol], and bromophenol blue [0.2% wt/vol]), then boiled for 10 min. Proteinase K (10 μL, 5 mg mL^−1^; Merck) was added, and the samples were incubated at 56 °C for 1 h. Following digestion, the samples were treated with water‐saturated phenol, vortexed briefly, and incubated at 68 °C for 30 min. Diethyl ether (1 mL) was then added, vortexed, and centrifuged (8800 × g, 15 min). The bottom layers were collected and diluted 1:10 with sample buffer, and 10 μL of the digested samples were loaded onto a 12% SDS–PAGE gel with a 5% stacking gel. Silver nitrate gel staining was used to visualize samples in the gel.

To isolate the lipid A, ≈1 mg of UCF_pc_ was washed with 500 μL of chloroform–methanol (1:2, vol/vol), followed by 500 μL of chloroform–methanol–water (3:2:0.25, vol/vol/vol) and several centrifugation steps (1200 × g, 30 min). Next, UCF_pc_ underwent a small‐scale extraction procedure.^[^ ^17^, ^37^ ^]^ Briefly, the washed UCF_pc_ was resuspended in 500 μL of isobutyric acid/1 M ammonium hydroxide (5:3, vol/vol) and heated at 100 °C for 1 h. After centrifugation (1200 × g, 30 min), the supernatant was collected, lyophilized, and washed multiple times with methanol, followed by a wash with chloroform–methanol–water (3:1.5:0.25, vol/vol). The resulting sample (LipA_sse_) was then prepared for chemical and MALDI‐TOF MS analyses. In parallel, supernatant from ultracentrifugation underwent reiterated ultracentrifugation steps and size‐exclusion chromatography on a Sephacryl High Resolution S‐100 (GE Healthcare) column, followed by check via SDS–PAGE. This sample was then used to stimulate HEK and THP cells along with isolated LPS. Finally, an aliquot of EPS‐EtOH (40 mg) was treated with acetate buffer (100 mM, pH 4.4.) and hydrolyzed at 100 °C for 2 h. Following the hydrolysis, methanol and chloroform were added to achieve a CH_3_OH/CHCl_3_/hydrolysate ratio of 2:2:1.8 (v/v/v). This mixture was shaken, centrifuged (4 °C, 8800 × g, 30 min), and then the chloroform phase was transferred to a clean tube and washed with methanol and water to reach a CHCl_3_/CH_3_OH/H_2_O ratio of 2:2:1.8.^[^ ^38^ ^]^ This washing procedure was repeated three times. Finally, the organic phases containing the lipid A fraction (LipA_abh_) were pooled, dried, and analyzed as described later.

##### Chemical Analyses

To analyze the fatty acids and monosaccharides present in the lipid A of *Polaribacter* sp. SM1127, both LipA_sse_ and LipA_abh_ were subjected to various treatments. First, an aliquot of each sample was methanolized using 1.25 M HCl in methanol at 85 °C for 16 h, followed by acetylation at 80 °C for 20 min.^[^ ^39^, ^40^ ^]^ Acting as such, sugars and lipids were detected via gas chromatography‐mass spectrometry (GC–MS) analysis of the related acetylated O‐methyl glycoside and methyl ester derivatives, respectively. In parallel, methanolysis in the same conditions as provided earlier was employed on another aliquot of both LipA_sse_ and LipA_abh_ followed by hexane extraction of the fatty acids which were then inspected by GC–MS. The fatty acid content was also determined by treating LipA_sse_ and LipA_abh_ with 4 M HCl at 100 °C for 4 h, followed by treatment with 5 M NaOH at 100 °C for 30 min. Fatty acids were then extracted in chloroform after adjusting the pH (≈3), methylated with diazomethane, and analyzed via GC–MS. O‐linked fatty acids were released using aqueous 0.5 M NaOH in methanol (1:1, vol/vol) at 85 °C for 2 h, followed by acidification (pH ≈ 3), chloroform extraction, and methylation with diazomethane, before GC–MS analysis. The absolute configuration of the 3‐hydroxy fatty acids was determined after their release using 4 M NaOH (100 °C, 4 h) and conversion to 3‐methoxy acid L‐phenylethylamides, which were then inspected by GC–MS.^[^ ^41^ ^]^ The retention times of the L‐phenylethylamides of known fatty acid standards were compared to those obtained from the lipid A samples. All compositional analyses were performed on an Agilent Technologies 7820 A Gas Chromatograph, equipped with a 5977B mass selective detector and an HP‐5 capillary column (30 m × 0.25 mm internal diameter, 1 mL min^−1^ flow rate, He as carrier gas).

##### MALDI‐TOF MS and MS/MS Analysis

MALDI‐TOF MS and MS/MS spectra were acquired in reflectron mode, negative ion polarity, using an ABSCIEX TOF/TOF 5800 mass spectrometer (Applied Biosystems, Foster City, CA, USA). The instrument was equipped with a neodymium‐doped yttrium aluminium garnet laser (*λ* = 349 nm), a 3 ns pulse width, and a repetition rate of up to 1000 Hz, along with delayed extraction technology. LipA_sse_ and LipA_abh_ were dissolved in a chloroform/methanol (50:50, vol/vol) mixture, while the matrix solution was 2,4,6‐trihydroxyacetophenone dissolved in methanol/0.1% trifluoroacetic acid/acetonitrile (7:2:1, vol/vol/vol) at a concentration of 75 mg mL^−1^.^[^ ^42^ ^]^ And, 0.5 μL of the sample and 0.5 μL of the matrix solution were applied to a stainless‐steel target plate and allowed to air dry at room temperature. For the MS analysis, each spectrum was generated by accumulating 2000 laser shots, while MS/MS spectra were obtained by summing 5000–7000 shots. Spots were randomly but evenly sampled and experiments were executed in triplicate.

##### HEK‐Blue hTLR4 and THP1‐Blue NF‐κB Cell Culture

HEK‐Blue hTLR4 cells (Invivogen, Toulouse, France) were grown in Dulbecco's modified Eagle's medium (Gibco, Thermo Fisher Scientific, Waltham, Massachusetts, United States) containing 4.5 g L^−1^ glucose and supplemented with 10% heat‐inactivated fetal bovine serum (FBS) (Gibco, Thermo Fisher Scientific, Waltham, Massachusetts, United States), 2 mM L‐glutamine (Gibco, Thermo Fisher Scientific, Waltham, Massachusetts, United States), 1% penicillin/streptomycin (Pen/Strep) (Gibco, Thermo Fisher Scientific, Waltham, Massachusetts, United States), and 100 μg mL^−1^ Normocin (Invivogen, Toulouse, France), as described previously.^[^ ^10^ ^]^ To ensure plasmid selection, HEK Blue selection (Invivogen, Toulouse, France) containing selection antibiotics was added to the medium following the instructions of the manufacturer.

THP1‐Blue NF‐κB cells (Invivogen, Toulouse, France) were grown in roswell park memorial institute 1640 medium (Gibco, Thermo Fisher Scientific, Waltham, Massachusetts, United States) containing 2 mM L‐glutamine (Gibco, Thermo Fisher Scientific, Waltham, Massachusetts, United States), 10% heat‐inactivated FBS (Gibco, Thermo Fisher Scientific, Waltham, Massachusetts, United States), 25 mM 4‐(2‐hydroxyethyl)‐1‐piperazineethanesulfonic acid (Gibco, Thermo Fisher Scientific, Waltham, Massachusetts, United States), 1% Pen/Strep (Gibco, Thermo Fisher Scientific, Waltham, Massachusetts, United States), and 100 μg mL^−1^ Normocin (Invivogen, Toulouse, France). To maintain plasmid selection, blasticidin (Invivogen, Toulouse, France) was used at a final concentration of 10 μg mL^−1^. All cell lines were maintained at 37 °C in a humidified atmosphere containing 5% CO_2_.

##### Stimulation Assays

HEK‐Blue hTLR4 were seeded into a 96‐well plate (3 × 10^4^ well^−1^). After allowing overnight attachment, HEK‐Blue hTLR4 cells were stimulated for 18 h with various concentrations (1‐10–100 ng mL^−1^) of *E. coli* O111:B4 LPS (*E. coli* LPS) (Invivogen, Toulouse, France) or *Polaribacter* sp. SM1127 LPS or EPS. THP1‐Blue NF‐κB cells were differentiated into macrophages according to the protocol reported by Baxter et al.^[^ ^43^ ^]^ Briefly, 60 × 10^3^ cells well^−1^ were seeded into a 96‐well plate. Cells were differentiated following treatment with 5 ng mL^−1^ phorbol 12‐myristate 13‐acetate (PMA) (Thermoscientific, Kandel, Germany) for 24 h. After PMA removal and washing, fresh PMA‐free medium was added to each well for a resting period of 72 h. The differentiated cells appeared in adhesion and were stimulated with various concentrations (10 and 100 ng mL^−1^) of *E. coli* LPS or *Polaribacter* sp. SM1127 LPS or EPS for 24 h to conduct immunological assays. Since HEK‐Blue hTLR4 and THP1‐Blue NF‐κB cells contain the SEAP reporter gene under the control of a promoter with NF‐kB binding sites, Quanti Blue assay (Invivogen, Toulouse, France) was performed on cell supernatants by evaluating SEAP activity as indicator of NF‐κB activation following LPS stimulation. Briefly, 20 μL of cell supernatant was added to 180 μL of Quanti‐blue solution, prepared according to the manufacturer's protocol (Invivogen, Toulouse, France). After 15 min of incubation at 37 °C, the absorbance at 620 nm was read using Tecan Infinite M Plex spectrophotometer (Tecan, Grödig, Austria).

##### Statistical Analysis

Absorbance values from unstimulated cells were subtracted and data are shown as mean  ± the standard deviation. Statistical analysis was performed using unpaired *t*‐test by GraphPad prism 8.0.1 software. A *p*‐value < 0.05 was considered statistically significant.

## Conflict of Interest

The authors declare no conflict of interest.

## Supporting information

Supplementary Material
