# Supplementary material for: Cold‐Adapted Lipid A from Polaribacter sp. SM1127: A Study of Structural Heterogeneity and Immunostimulatory Properties
Source: Chembiochem. 2025 Apr 21;26(12):e202500100. doi: 10.1002/cbic.202500100 (PMC12177697; doi:10.1002/cbic.202500100)
Supplement: Supplementary file 1 — Supplementary Material [file CBIC-26-e202500100-s001.pdf]

Supporting Information

Cold-Adapted Lipid A from Polaribacter sp. SM1127: A Study of Structural Heterogeneity and Immunostimulatory properties

Roberta Cirella, Emanuela Andretta, Luca De Simone Carone, Francesca Olmeo, Mei-Ling Sun, Yu-Zhong Zhang, Marcello Mercogliano, Antonio Molinaro, Alba Silipo, Flaviana Di Lorenzo

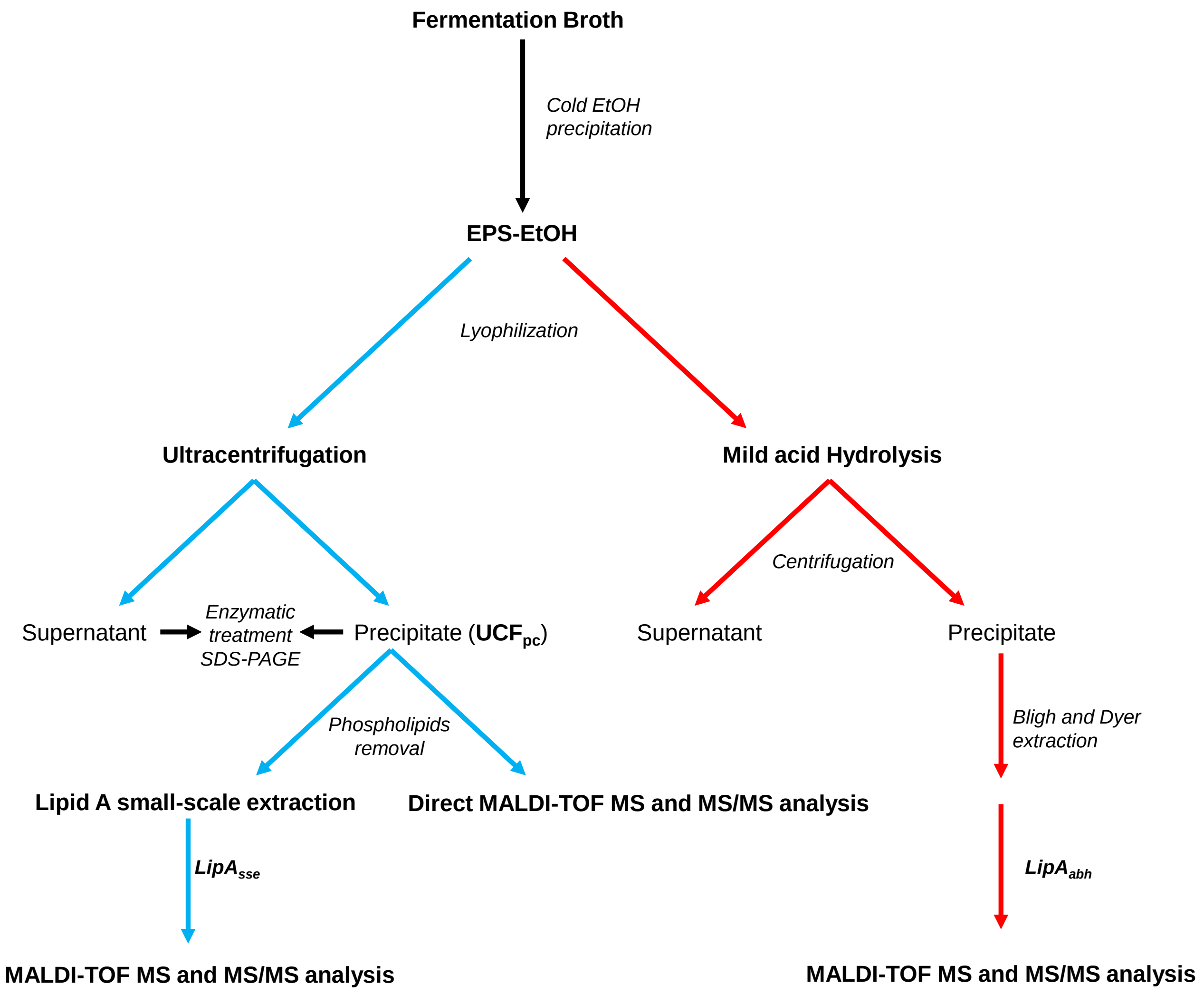

Figure S1. A diagram illustrating the methodology used to extract, identify, and characterize the lipid A from *Polaribacter* sp. SM1127, beginning with the crude ethanol-precipitate (*EPS-EtOH*) that was initially collected for isolating the EPS.
